# Supplementary material for: Alternative platelet differentiation pathways initiated by nonhierarchically related hematopoietic stem cells
Source: Nat Immunol. 2024 May 30;25(6):1007–19. doi: 10.1038/s41590-024-01845-6 (PMC11147777; doi:10.1038/s41590-024-01845-6)
Supplement: Supplementary file 1 — Supplementary Figs. 1–4 and Tables 1–3 and 5. [file 41590_2024_1845_MOESM1_ESM.pdf]

nature immunology

Supplementary information https://doi.org/10.1038/s41590-024-01845-6

Alternative platelet differentiation pathways initiated by nonhierarchically related hematopoietic stem cells

In the format provided by the authors and unedited

Alternative platelet differentiation pathways initiated by non-hierarchically related hematopoietic stem cells

Supplementary Information

Supplementary Fig. 1. Quality controls of RNA sequencing data and assessment and correction of batch effects.

Supplementary Fig. 2. UMAP distribution of biological replicates before and after batch correction.

Supplementary Fig. 3. MkPs rapidly replenished by HSCs in steady-state show upregulation of genes upregulated in MkPs derived from *Vwf*-tdTomato$^+$ P-HSCs.

Supplementary Fig. 4. Validation of cell lineage identity of reconstituted blood cells defined as myeloid by flow cytometry analysis.

Supplementary Table 1. Secondary transplantation of donor-derived *Vwf*-tdTomato$^-$ cells sorted from primary recipients of single *Vwf*-tdTomato$^-$ Multi-HSC from which *Vwf*-tdTomato$^+$ cells were not sortable.

Supplementary Table 2. Secondary transplantation of donor-derived *Vwf*-tdTomato$^+$ and *Vwf*-tdTomato$^-$ cells sorted from primary recipients of single *Vwf*-tdTomato$^-$ Multi-HSC.

Supplementary Table 3. Secondary transplantation of donor-derived *Vwf*-tdTomato$^+$ and *Vwf*-tdTomato$^-$ cells sorted from primary recipients of single *Vwf*-tdTomato$^+$ P-HSC.

Supplementary Table 4 (spreadsheet file with 10 tabs). Gene expression in cells derived from single transplanted *Vwf*-tdTomato$^+$ P-HSCs compared to cells derived from single *Vwf*-tdTomato$^-$ Multi-HSCs. "MolO HSC DEGs": differentially expressed genes (combined Wilcoxon/Fishers' exact test) in molecular HSCs. "tradeSeq trajectory DEGs": differentially expressed genes in total cells along pseudotime. "tradeSeq random variable genes": list of genes used for the background correlation in **Fig. 3k**. "MkP DEGs": differentially expressed genes (combined Wilcoxon/Fishers' exact test) in molecular MkPs. "P-MkP v CD48+ Multi-MkP": differentially expressed genes in CD48$^-$ P-MkPs compared to CD48$^+$ Multi-MkPs, based on cell surface protein detection by flow cytometry. "P-MkP v CD48- Multi-MkP": differentially expressed genes in CD48$^-$ P-MkPs compared to CD48$^+$ Multi-MkPs, based on cell surface protein detection by flow cytometry. "RNA P-MkP v Cd48+ Multi-MkP": differentially expressed genes in P-MkPs negative for *Cd48* transcript compared to Multi-MkPs positive for *Cd48* transcript. "RNA P-MkP v Cd48- Multi-MkP": differentially expressed genes in P-MkPs negative for *Cd48* transcript compared to Multi-MkPs negative for *Cd48* transcript. "RNA Cd48+ v Cd48- Multi-MkP": differentially expressed genes in Multi-MkPs positive for *Cd48* transcript compared to Multi-MkPs negative for *Cd48* transcript. Combined Wilcoxon/Fishers' exact test used for identification of differential expressed genes.

Supplementary Table 5. Monoclonal antibodies and staining dyes used in flow cytometry analysis and cell sorting. Order of lot number and dilution used are matched. For some antibodies different dilutions were used for Bone Marrow (BM) and Peripheral Blood (PB), as indicated.

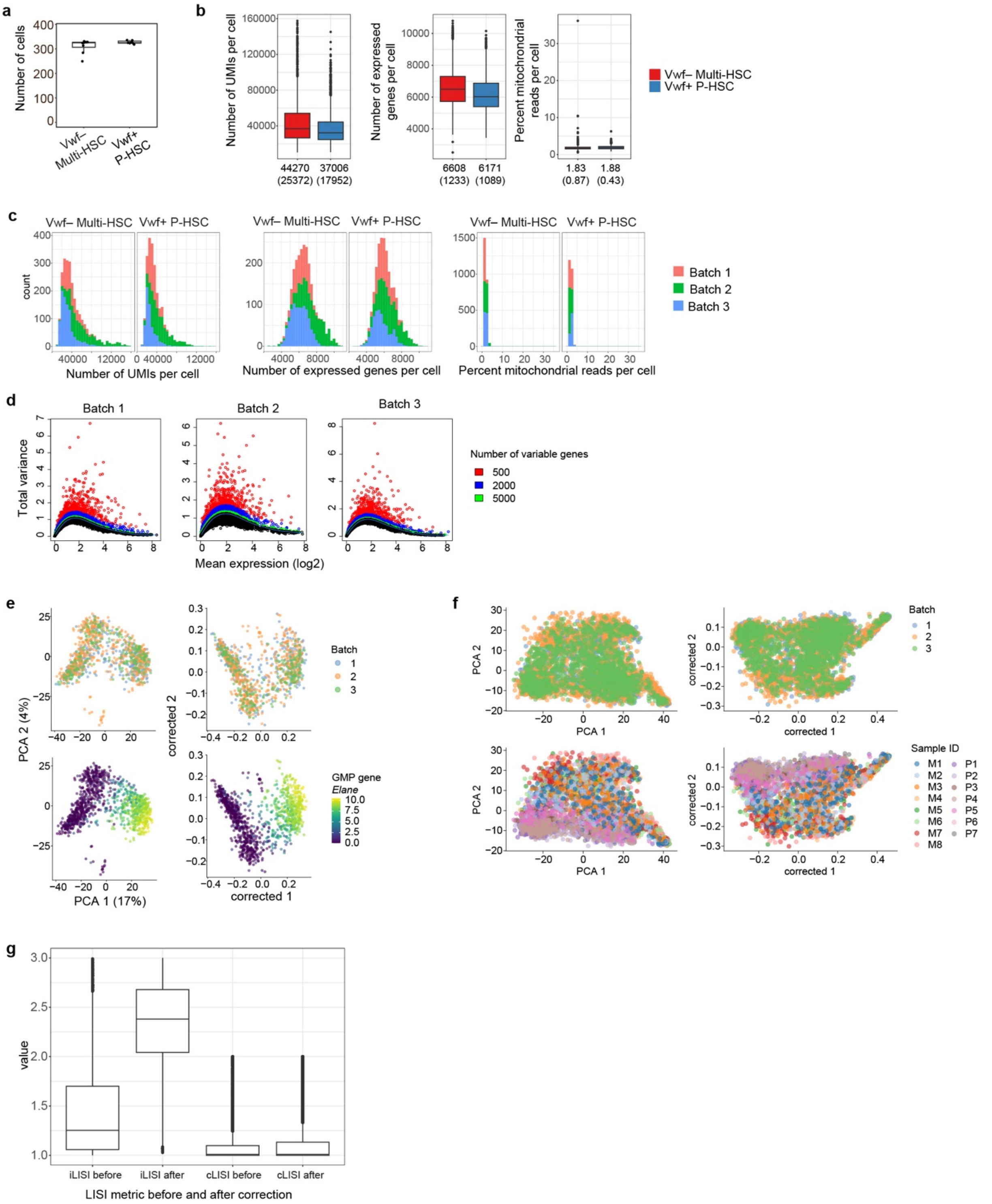

Supplementary Fig. 1. Quality controls of RNA sequencing data and assessment and correction of batch effects.

a, Number of cells included in the RNA sequencing analysis from individual mice reconstituted by single transplanted *Vwf*-tdTomato^{+} P-HSCs (n=7) and *Vwf*-tdTomato^{-} Multi-HSCs (n=8). Centre line: median; box limits: first and third quartiles; whiskers: 1.5*IQR; dots represent individual mice.

b, Box plots showing distribution of UMI counts (left), number of expressed genes detected (middle) and percent mitochondrial RNA (right) in cells replenished by single *Vwf*-tdTomato^{-} Multi-HSCs (n=2478 cells) and *Vwf*-tdTomato^{+} P-HSCs (n=2290 cells). Centre line: median;

box limits: first and third quartiles; whiskers: 1.5*IQR; dots: individual outlier cells. Numbers below each plot indicate the mean value with standard deviations in parenthesis.
c, Distribution of UMI counts (left plots), number of expressed genes detected (middle plots), and percent mitochondrial RNA (right plots) in cells originating from *Vwf*-tdTomato^{-} Multi-HSCs (left side) and *Vwf*-tdTomato^{+} P-HSCs (right side).
d, Selection of variable genes. X-axis: Mean log expression, y-axis: total variance. Top 500, 2000, and 5000 genes with the highest biological variance are highlighted by the color scale.
e, Batch effect analysis, using the mutual nearest neighbour approach, of three independent sequencing runs (1-3) of control granulocyte/macrophage progenitor (GMP) and LSK cells sorted from the same pool of BM cells derived from three wildtype steady-state mice. These control cells were included in all experiments and in all plates with single cells sorted from mice reconstituted by *Vwf*-tdTomato^{-} Multi-HSCs and *Vwf*-tdTomato^{+} P-HSCs. Top row: uncorrected (left) and batch-corrected (right) Principal Component Analysis (PCA) plots. Bottom row: expression of *Elane*, a GMP marker, within the uncorrected (left) and batch-corrected (right) PCA plots.
f, Batch effect analysis (as in **e**) applied to all cells sorted from mice reconstituted by *Vwf*-tdTomato^{-} Multi-HSCs (M1-8) or *Vwf*-tdTomato^{+} P-HSCs (P1-7) based on sequencing runs. Cells are colored by batch in the top row and by mouse ID in the bottom row.
g, Batch integration evaluation using iLISI and cLISI metrics before and after correction. Centre line: median; box limits: first and third quartiles; whiskers: 1.5*IQR; dots: outliers (n=4768 cells).

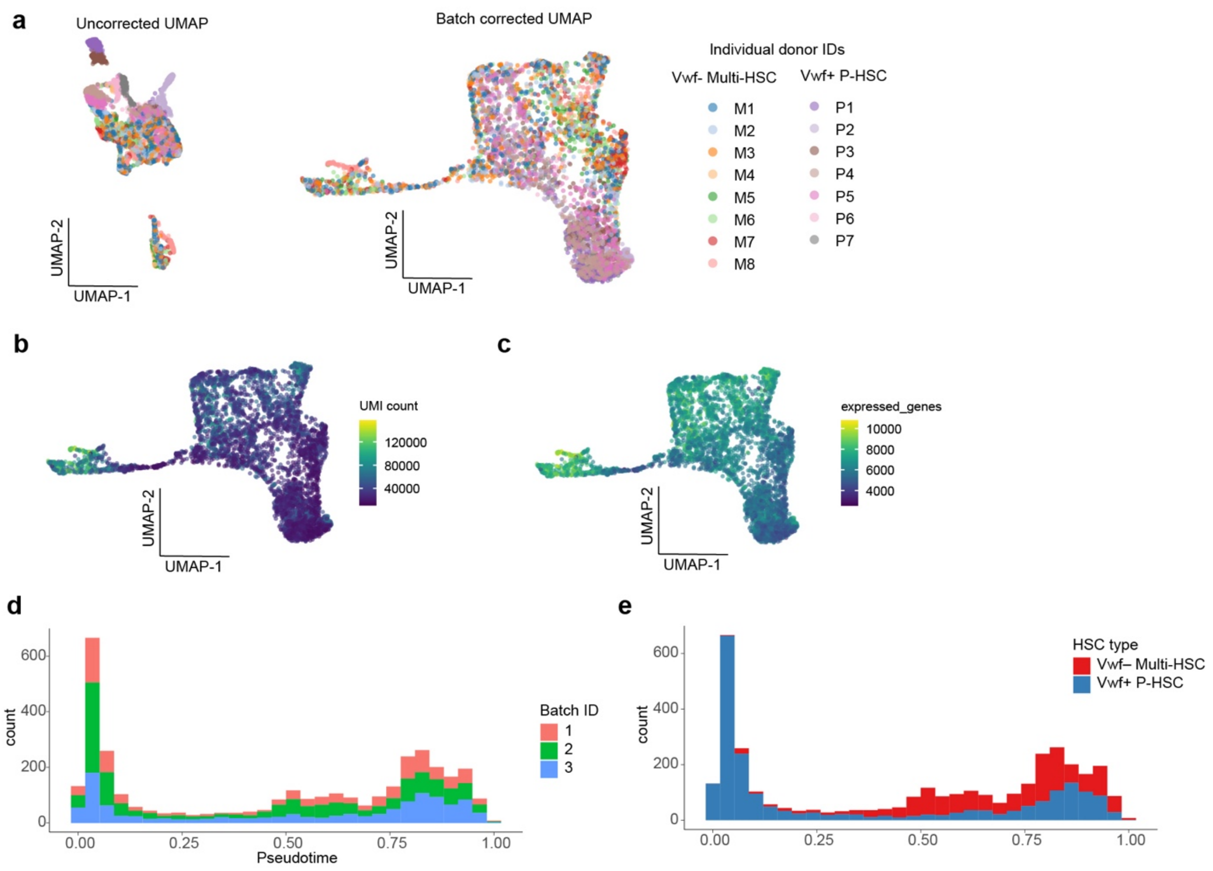

Supplementary Fig. 2. UMAP distribution of biological replicates before and after batch correction.

a, Distribution of all cells originating from donor *Vwf*-tdTomato^{-} Multi-HSC (M1-8) or *Vwf*-tdTomato^{+} P-HSC (P1-7) displayed in uncorrected and batch-corrected UMAP plots.

b-c, UMAP distribution of UMI counts (**b**) and number of detected genes (**c**).

d-e, Distribution of cells along pseudotime based on sequencing batch (**d**) and HSC type (**e**).

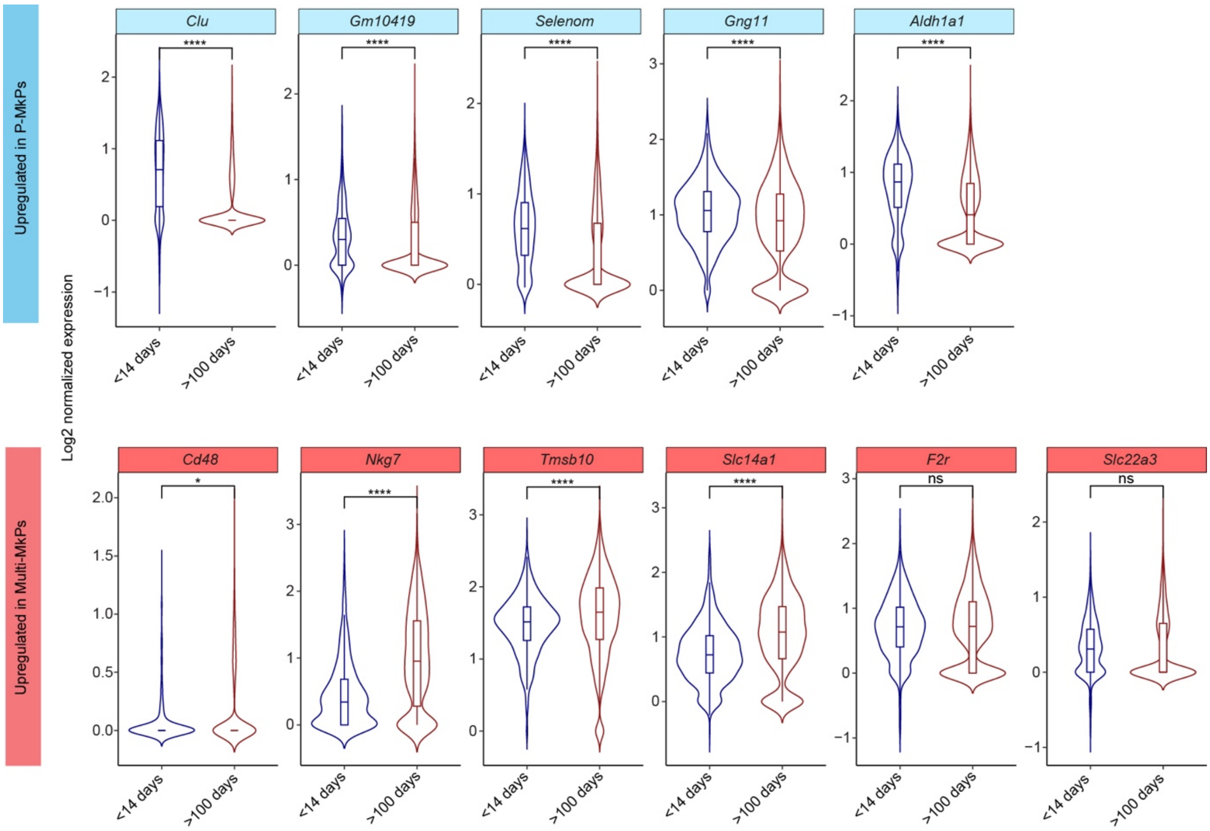

Supplementary Fig. 3. MkPs rapidly replenished by HSCs in steady-state show upregulation of genes upregulated in MkPs derived from *Vwf*-tdTomato⁺ P-HSCs.
Reanalysis of published data set of single cell RNA expression (Kucinski *et al*, Cell Stem Cell 2024). The indicated genes were investigated in molecularly defined MkPs ("Meg prog") labelled in steady-state *Hoxb5*-Cre^{ERT2}-$R26^{Tom}$ reporter mice before 14 days (n=747 cells; blue) or more than 100 days (n=3014 cells; red) after Tamoxifen induction of tdTomato labelling specifically within the *Hoxb5*⁺ HSC compartment. Violin plots overlaid with box plots show normalized expression of the genes selected based on being the most up- or down-regulated in molecularly defined MkPs (**Fig. 4a**, **Supplementary Table 4**) replenished by *Vwf*-tdTomato⁺ P-HSCs (P-MkPs) when compared to MkPs replenished by *Vwf*-tdTomato⁻ Multi-HSCs (Multi-MkPs). For further details see **Methods**. Lines within boxes indicate median values, boxes represent the first and third quartiles, whiskers indicate 1.5*IQR. Significance levels are denoted as * for $p < 0.05$, **** for $p < 0.0001$ and ns for non-significance ($p \geq 0.05$), determined by the one-sided Wilcoxon test with adjustment by the Benjamini-Hochberg method. *P* values: *Clu*=2.44×10^{-137}, *Gm10419*=6.29×10^{-24}, *Selenom*=7.15×10^{-79}, *Gng11*=1.06×10^{-12}, *Aldh1a1*=4.37×10^{-60}, *Cd48*=0.049, *Nkg7*=6.74×10^{-64}, *Tmsb10*=2.49×10^{-13}, *Slc14a1*=4.75×10^{-42}, *F2r*=0.82, *Slc22a3*=1.00.

a

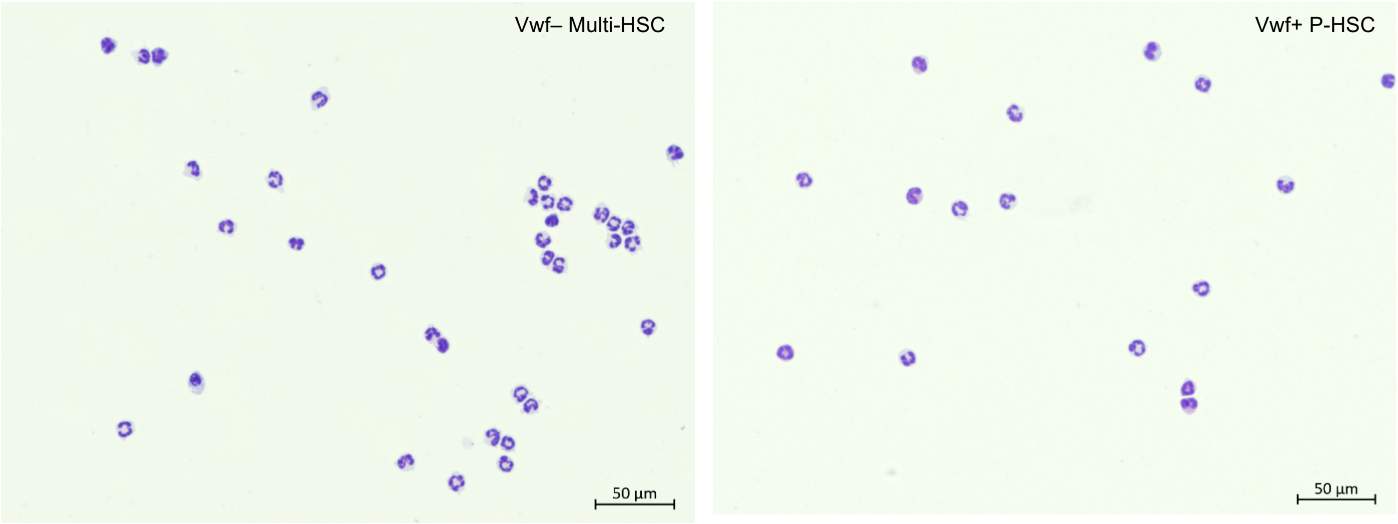

b

	Multi-HSC 1	Multi-HSC 2	P-HSC 1	P-HSC 2
Granulocytes	78	88	80	91
Monocytes	22	12	20	9
Erythrocytes	0	0	0	0
Lymphocytes	0	0	0	0

Supplementary Fig. 4. Validation of cell lineage identity of reconstituted blood cells defined as myeloid by flow cytometry analysis.

a. Representative cytospin images following staining with Eosin-Y/Azure-A/Methylene-Blue of flow cytometry purified CD11b$^+$NK1.1$^-$CD19$^-$CD4/CD8a$^-$CD45.1$^-$CD45.2$^+$ blood cells from CD45.1 recipient mice reconstituted by a single transplanted CD45.2 LSK*Gata1*-eGFP$^-$ CD34$^-$CD150$^+$CD48$^-$CD201$^+$ *Vwf*-tdTomato$^-$ Multi-HSC (left, n=2) or *Vwf*-tdTomato$^+$ P-HSC (right, n=2).

b. Differential counts of 100 cells per mouse, demonstrating that cells with the cell surface phenotype of CD11b$^+$NK1.1$^-$CD19$^-$CD4/CD8a$^-$CD45.1$^-$CD45.2$^+$ represent pure myeloid (granulocyte/ monocyte) cells.

Supplementary Table 1 | Secondary transplantation of donor-derived *Vwf*-tdTomato^{-} cells sorted from primary recipients of single *Vwf*-tdTomato^{-} Multi-HSC from which *Vwf*-tdTomato^{+} cells were not sortable.

	Secondary recipients			
Primary recipients	CD45.2^{+}CD45.1^{-} population transplanted	Cells per recipient	Mice	Reconstitution at 16-18 weeks
Multi-HSC #1	LSKCD150^{+}CD48^{-} *Vwf*-tdTomato^{-}	132	1	Multilineage, platelet/erythroid/myeloid bias
Multi-HSC #2	LSKCD150^{+}CD48^{-} *Vwf*-tdTomato^{-}	180	1	Multilineage, no bias
			2	Multilineage, lymphoid-bias
Multi-HSC #3	LSKCD150^{+}CD48^{-} *Vwf*-tdTomato^{-}	95	1	Multilineage, no bias
Multi-HSC #4	LSK*Gata1*eGFP^{-}CD150^{+}CD48^{-} CD201^{+}*Vwf*-tdTomato^{-}	90	1	Multilineage no bias
			2	Lymphoid/erythroid only
	LSK*Gata1*eGFP^{-}CD150^{+}CD48^{-} CD201^{-}*Vwf*-tdTomato^{-}	110	1	Lymphoid only
			2	Lymphoid only

Related to **Fig. 2c**. All sortable donor-derived LSKCD150^{+}CD48^{-}CD45.1^{-}CD45.2^{+}*Vwf*-tdTomato^{-} cells were transplanted from primaries #1-3 and all sortable donor-derived LSK*Gata1*eGFP^{-}CD150^{+}CD48^{-}CD45.1^{-}CD45.2^{+} CD201^{+}*Vwf*-tdTomato^{-} and CD201^{-}*Vwf*-tdTomato^{-} cells were transplanted from primary #4. Simultaneous sorting of *Vwf*-tdTomato^{+} cells was attempted in all cases, but none were sortable. For primary #4, only the transplantation of CD201^{+}*Vwf*-tdTomato^{-} cells is included in the figure.

Supplementary Table 2 | Secondary transplantation of donor-derived *Vwf*-tdTomato^{+} and *Vwf*-tdTomato^{-} cells sorted from primary recipients of single *Vwf*-tdTomato^{-} Multi-HSC.

	Secondary recipients			
Primary recipients	CD45.2^{+}CD45.1^{-} population transplanted	Cells per recipient	Mice	Reconstitution at 16-18 weeks
Multi-HSC #5	LSK *Vwf*-tdTomato^{+}	100	1	None
			2	Lymphoid only
			3	Lymphoid only
	LSK *Vwf*-tdTomato^{-}	3400	1	Multilineage, no bias
			2	Multilineage, no bias
			3	Multilineage, no bias
Multi-HSC #6	LSK *Vwf*-tdTomato^{+}	55	1	None
			2	None
			3	Lymphoid only
	LSK *Vwf*-tdTomato^{-}	3300	1	Multilineage, lymphoid bias
			2	Multilineage, no bias
			3	Multilineage, lymphoid bias
Multi-HSC #7	LSK *Vwf*-tdTomato^{+}	7	1	None
	LSK *Vwf*-tdTomato^{-}	500	1	Multilineage, lymphoid bias
			2	Multilineage, lymphoid bias
			3	Multilineage, lymphoid bias
Multi-HSC #8	LSKCD150^{+}CD48^{-} *Vwf*-tdTomato^{+}	1	1	None
	LSKCD150^{+}CD48^{-} *Vwf*-tdTomato^{-}	115	1	Multilineage, lymphoid bias
Multi-HSC #9	LSKCD150^{+}CD48^{-} *Vwf*-tdTomato^{+}	4	1	None
	LSKCD150^{+}CD48^{-} *Vwf*-tdTomato^{-}	391	1	Multilineage, no bias
			2	Multilineage, no bias
Multi-HSC #10	LSKCD150^{+}CD48^{-} *Vwf*-tdTomato^{+}	5	1	None
	LSKCD150^{+}CD48^{-} *Vwf*-tdTomato^{-}	444	1	Multilineage, no bias
			2	Multilineage, no bias
Multi-HSC #11	LSKCD150^{+}CD48^{-} *Vwf*-tdTomato^{+}	1	1	None
	LSKCD150^{+}CD48^{-} *Vwf*-tdTomato^{-}	480	1	Multilineage, no bias
			2	Multilineage, no bias

Related to **Fig. 2d**. All sortable donor-derived LSKCD45.1^{-}CD45.2^{+}*Vwf*-tdTomato^{+} cells were transplanted from primaries #5-7 and then the number of *Vwf*-tdTomato^{-} cells to transplant was calculated to match the ratio between LSKCD150^{+}CD48^{-}CD45.1^{-}CD45.2^{+}*Vwf*-tdTomato^{+} and LSKCD150^{+}CD48^{-}CD45.1^{-}CD45.2^{+}*Vwf*-tdTomato^{-} cells present in each of these primaries. All sortable donor-derived LSKCD150^{+}CD48^{-}CD45.1^{-}CD45.2^{+}*Vwf*-tdTomato^{+} and *Vwf*-tdTomato^{-} cells were transplanted from primaries #8-11.

Supplementary Table 3 | Secondary transplantation of donor-derived *Vwf*-tdTomato^{+} and *Vwf*-tdTomato^{-} cells sorted from primary recipients of single *Vwf*-tdTomato^{+} P-HSC.

	Secondary recipients			
Primary recipients	CD45.2^{+}CD45.1^{-} population transplanted	Cells per recipient	Mice	Reconstitution at 16-18 weeks
P-HSC #1	LSKCD150^{+}CD48^{-} *Vwf*-tdTomato^{+}	11676	1	Platelet/erythroid/myeloid only
			2	Platelet/erythroid/myeloid only
	LSKCD150^{+}CD48^{-} *Vwf*-tdTomato^{-}	208	1	None
			2	None
P-HSC #2	LSKCD150^{+}CD48^{-} *Vwf*-tdTomato^{+}	1542	1	Platelet/erythroid/myeloid only
			2	Platelet/erythroid/myeloid only
	LSKCD150^{+}CD48^{-} *Vwf*-tdTomato^{-}	61	1	None
P-HSC #3	LSK*Gata1*eGFP^{-}CD150^{+}CD48^{-} CD201^{+}*Vwf*-tdTomato^{+}	219	1	Platelet only
			2	Platelet/erythroid/myeloid only
	LSK*Gata1*eGFP^{-}CD150^{+}CD48^{-} CD201^{-}*Vwf*-tdTomato^{+}	8	1	None
			2	None
	LSK*Gata1*eGFP^{-}CD150^{+}CD48^{-} CD201^{+}*Vwf*-tdTomato^{-}	24	1	None
			2	None
	LSK*Gata1*eGFP^{-}CD150^{+}CD48^{-} CD201^{-}*Vwf*-tdTomato^{-}	2	1	None
			2	None
P-HSC #4	LSK*Gata1*eGFP^{-}CD150^{+}CD48^{-} CD201^{+}*Vwf*-tdTomato^{+}	379	1	Platelet/erythroid/myeloid only
			2	Platelet/erythroid/myeloid only
	LSK*Gata1*eGFP^{-}CD150^{+}CD48^{-} CD201^{-}*Vwf*-tdTomato^{+}	22	1	Platelet only
			2	Platelet/erythroid/myeloid only
	LSK*Gata1*eGFP^{-}CD150^{+}CD48^{-} CD201^{+}*Vwf*-tdTomato^{-}	749	1 (*)	Platelet/erythroid/myeloid only
	LSK*Gata1*eGFP^{-}CD150^{+}CD48^{-} CD201^{-}*Vwf*-tdTomato^{-}	440	1 (*)	Platelet/erythroid only

Related to **Fig. 2e**. All sortable donor-derived LSKCD150^{+}CD48^{-}CD45.1^{-}CD45.2^{+}*Vwf*-tdTomato^{+} and *Vwf*-tdTomato^{-} cells were transplanted from primaries #1-2. All sortable donor-derived LSKCD150^{+}CD48^{-}CD45.1^{-}CD45.2^{+} CD201^{+}*Vwf*-tdTomato^{+}, CD201^{-}*Vwf*-tdTomato^{+}, CD201^{+}*Vwf*-tdTomato^{-}, and CD201^{-}*Vwf*-tdTomato^{-} cells were transplanted from primaries #3-4. For primary #3, only the transplantation of CD201^{+}*Vwf*-tdTomato^{-} and CD201^{+}*Vwf*-tdTomato^{+} cells is included in the figure. Primary #4 is represented in **Extended Data Fig. 1f**. (*) Two recipients were transplanted for the marked populations, but only one of each survived until the final time point for analysis of blood replenishment.

Supplementary Table 4

Related to **Fig. 3f, 3j, 3k, 4a, 5a, 6a, 6b, 6c, 6d**.

Supplementary Table 5 | Monoclonal antibodies and staining dyes used in flow cytometry analysis and cell sorting.

Marker/dye/reagent	Clone	Conjugate	Supplier	Catalog Nb	Lot	Dilution	Panels
4’,6-Diamidino-2-Phenylindole, Dilactate (DAPI)	-	-	Invitrogen	D3571	1387190	50000	WBC 2, 5, 6; BM 6, 11
7-Aminoactinomycin D (7-AAD)	-	-	Cayman Chemical, Sigma-Aldrich	11397	0519011-4	100	WBC 1, 3, 4; BM 1-5, 7-10, 12-17
B220 (CD45R)	RA3-6B2	BUV395	BD	563793	121784	600	BM 17-19
		PE-Cy5	BioLegend	103210	B182591, B247904	800, 1200	BM 1-16, 20
CD4	RM4-5	APC	BioLegend	100516	B277608	1600	WBC 3
		APC-eF780	eBioscience	47-0042-82	4278618	1600	WBC 2, 4-7
		BUV395	BD	740208	8003857	800	BM 18
		BV650	BioLegend	100546	B238102	200	WBC 1
		PE-Cy5	BioLegend	100514	B205393	2000	BM 1-9, 15-16, 20
CD5	53-7.3	BUV395	BD	740206	7300709, 7300710, 1155041	300, 300, 300	BM 17-19
		PE-Cy5	BioLegend	100610	B178256	1200	BM 1-16, 20
CD8a	53-6.7	APC-eF780	eBioscience	47-0081-82	2011697, 2311247	1200, 1200	WBC 1-7
		BUV395	BD	563786	7249930, 91417	600, 600	BM 17-19
		PE-Cy5	BioLegend	100710	B187949, B300603	1200, 1200	BM 1-16, 20
CD9	KMC8	BV421	BD	752985	3333288	100	BM 20
CD11b (Mac-1)	M1/70	APC	BioLegend	101212	B188460, B203536	1200, 600	WBC 2, 4-7
		PE-Cy5	BioLegend	101210	B197820	1000	BM 1-9, 15-16, 20
		PE-Cy7	BioLegend	101216	B227804	1600	WBC 1, 3
CD16/32	93	BV510	BioLegend	101333	B303788	200	BM 14, 17
CD19	eBio1D3	eF450	eBioscience	48-0193-82	E10916-1632	200	WBC 1, 3, 4
		PE-Cy5	eBioscience	15-0193-83	E06109-1631	1200	WBC 2, 5
		PE-Cy7	eBioscience	25-0193-82	E07526-1635	1000	WBC 6
CD24	30-F1	BUV737	BD	752766	3333286	100	BM 20
CD34	RAM34	AF700	eBioscience, BD	56-0341-82	7136967, 4324661, 2267417	10, 15, 10	BM 1-9
CD41	MWReg30	BV421	BioLegend	133912	B358797	200	P/E 3
		BV605	BD	747728	7117619, 163927	100, 100	BM 14
		BV650	BD	740504	1032384, 329110, 1329728	150, 150, 150	WBC 6; BM 17; 19, 20
		PE-Cy7	eBioscience	25-0411-82	2158708	800	P/E 1, 2, 4; WBC 2, 4, 5, 7
			Biolegend	133916	B293729, B247402, B203702, B283815	400, 200, 200, 200	P/E 1, 2, 4; WBC 2, 4, 5, 7
CD45.1	A20	BUV395	BD	565212	7122534, 9267616, B323829,1187834	PB 200- BM 50, PB 200- BM 50, PB 200- BM 50, PB 200- BM 50	WBC 2; BM 13-14, 20
		BV605	BioLegend	110738	B303397	100	WBC 1
		BV650	BioLegend	110735	1032384	150	WBC 3, 4; BM 10-12, 15-16
CD45.2	104	AF700	BioLegend	109822	B312840, B295392	100, 50	WBC 1, 3, 4; BM 10-16, 19, 20
		BUV737	BD	564880	7122542, 9186051	200, 400	WBC 2
CD48	HM48-1	APC	BioLegend	103412	B181146	600	BM 1, 4, 6, 7, 10-11, 14-16, 19, 20
		BV510	BD	563536	2075366	300	BM 2, 8
		PE-Cy7	BioLegend	103424	B375105	150	BM 3, 9, 12
		BUV737	BD	749666	1112972, 1187532, 1187535, 8212982, 1187533, 1112972, 1187536, 1322018	50, 50, 50, 100, 50, 100, 50, 50	BM 5, 13, 17, 18
CD49b	HMa2	BV711	Thermo Fisher	740704	1096977	800	BM 20
CD105 (Endoglin)	MJ7/18	BUV737	BD	741873	59053, 8958, 59046	200, 100, 100	BM 14, 19
		BV786	BD	564746	9253273, 1095007	100, 200	BM 17
CD117 (c-Kit)	2B8	APC-eF780	eBioscience	47-1171-82	E08461-1638, 2018834	1600, 1600	BM 1-20
CD135 (Flt3)	A2F10	Biotin	BioLegend	135308	E02732-1631	400	BM 16, 18
		BV421	BioLegend	135315	B329451, B314867, B342433, B373525, B268954, B278023	35, 50, 35, 35, 20, 20	BM 5, 7-10, 12-15, 17
CD150	TC15-12F12.2	APC	BioLegend	115910	B283740	100	P/E 1
		BV785	BioLegend	115937	B283815	PB 400- BM 200	P/E 2, 3, 4; BM 3, 4, 6-9, 11, 12, 14, 18-20
		PE-Cy7	BioLegend	115914	B190180	600	BM 1, 2, 5, 10, 13, 15-17
CD201 (EPCR)	eBio1560	APC	eBioscience	17-2012-82	2162235, 2345025	200, 200	BM 2, 3, 5, 8, 9, 12, 13, 17, 18
GR-1 (Ly-6G/Ly-6C)	RB6-8C5	BUV395	BD	563849	7349817, 2245850	800, 2000	BM 17-19
		Pacific Orange	Invitrogen	RM3030	1660881, 1860716	400, 300	WBC 4-7
		PE-Cy5	BioLegend	108410	B179158	1500	BM 1-16, 20
NK1.1 (CD161)	PK136	PE-Cy5	BioLegend	108716	B242312	400	WBC 1, 3, 4
		Pacific Blue	BioLegend	108722	B296431, B318741, B224552, B365322	1000, 1000, 600, 1000	WBC 2, 5-7
SCA-1 (Ly-6A/E)	D7	BV605	BioLegend	108133	B219335, B301237, B323829	100, 100, 100	BM 1, 2, 3, 5, 10, 12, 13
		BV650	BioLegend	108143	B309533	400	BM 9
		BV786	BD	563991	8352656	300	BM 15-16
	E13-161.7	FITC	BioLegend	122506	B163258	500	BM 17
		PE-Cy7	BioLegend	122514	B194434	800	BM 4, 6-8, 11, 14, 18-20
Streptavidin	-	BV421	BioLegend	405226	B316686	100	BM 16
		BB515	BD	564453	6175654	200	BM18
TER119	TER-119	APC	BioLegend	116212	B246740	600	P/E 2, 3, 4
			eBioscience	17-5921-81	2151462	400	P/E 2, 3, 4
		BUV395	BD	563827	7143754, 7235927, 1054261	200, 200, 400	BM 17-19
		PE-Cy5	BioLegend	116210	B169021, B208715, B277009	600, 600, 600	BM 1-16, 20
		PerCP-Cy5.5	eBioscience	45-5921-82	2102848	200	P/E 1
Retic-Count Reagent (Thiazole Orange)	-	-	BD	349204	1305842	-	P/E 4

P/E 1-2, WBC 1-2: Reconstitution analysis of platelets and erythrocytes (P/E) and white blood cells (WBC) after transplantation from *Vwf*-tdTomato/*Gata1*-eGFP donors.
P/E 3, WBC 3: Reconstitution analysis of P/E and WBC after transplantation from *Flt3*Cre donors.
P/E 3, WBC 4: Analysis of P/E and WBC of steady-state *Flt3*Cre mice.
P/E 4, WBC 5-6: Analysis of P/E and WBC before and after Cyclophosphamide (CP) and treatment of *Flt3*Cre and *Vav*Cre mice.
P/E 4, WBC 7: Analysis of P/E and WBC before and after 5-Fluorouracil (5FU) treatment of *Flt3*Cre mice.
P/E 4: Analysis of P/E before and after anti-CD42b antibody (αCD42b) treatment of *Flt3*Cre mice.
BM 1-5: Single-HSC sorting from *Vwf*-tdTomato/*Gata1*-eGFP donors.
BM 6-9: Single-HSC sorting from *Flt3*Cre donors.
BM 10-13: Analysis of reconstituted mice and LSK/HSC sorting for secondary transplantation (*Vwf*-tdTomato/*Gata1*-eGFP primary donors).
BM 14: Analysis of reconstituted mice and LSK/LK sorting for RNA sequencing (*Vwf*-tdTomato/*Gata1*-eGFP primary donors).
BM 15-16: Analysis of reconstituted mice (*Flt3*Cre primary donors).
BM 17: Analysis of *Flt3*Cre mice before and after CP treatment.
BM 18: Analysis of *Flt3*Cre mice before and after 5FU treatment.
BM 19: Analysis of wild-type mice before and after αCD42b treatment.
BM 20: Analysis of reconstituted mice for MkP immunophenotype (*Vwf*-tdTomato/*Gata1*-eGFP primary donors).
