# Supplementary material for: Alternative platelet differentiation pathways initiated by nonhierarchically related hematopoietic stem cells
Source: Nat Immunol. 2024 May 30;25(6):1007–19. doi: 10.1038/s41590-024-01845-6 (PMC11147777; doi:10.1038/s41590-024-01845-6)
Supplement: Supplementary file 2 — Reporting Summary [file 41590_2024_1845_MOESM2_ESM.pdf]

nature portfolio

Corresponding author(s): Joana Carrelha, Sten Eirik W. Jacobsen

Last updated by author(s): Apr 8, 2024

Reporting Summary

Nature Portfolio wishes to improve the reproducibility of the work that we publish. This form provides structure for consistency and transparency in reporting. For further information on Nature Portfolio policies, see our Editorial Policies and the Editorial Policy Checklist.

Statistics

For all statistical analyses, confirm that the following items are present in the figure legend, table legend, main text, or Methods section.

n/a	Confirmed	
☐	☒	The exact sample size (*n*) for each experimental group/condition, given as a discrete number and unit of measurement
☐	☒	A statement on whether measurements were taken from distinct samples or whether the same sample was measured repeatedly
☐	☒	The statistical test(s) used AND whether they are one- or two-sided<br>*Only common tests should be described solely by name; describe more complex techniques in the Methods section.*
☐	☒	A description of all covariates tested
☐	☒	A description of any assumptions or corrections, such as tests of normality and adjustment for multiple comparisons
☐	☒	A full description of the statistical parameters including central tendency (e.g. means) or other basic estimates (e.g. regression coefficient) AND variation (e.g. standard deviation) or associated estimates of uncertainty (e.g. confidence intervals)
☐	☒	For null hypothesis testing, the test statistic (e.g. *F*, *t*, *r*) with confidence intervals, effect sizes, degrees of freedom and *P* value noted<br>*Give P values as exact values whenever suitable.*
☒	☐	For Bayesian analysis, information on the choice of priors and Markov chain Monte Carlo settings
☒	☐	For hierarchical and complex designs, identification of the appropriate level for tests and full reporting of outcomes
☐	☒	Estimates of effect sizes (e.g. Cohen's *d*, Pearson's *r*), indicating how they were calculated

Our web collection on statistics for biologists contains articles on many of the points above.

Software and code

Policy information about availability of computer code

Data collection

BD FACSDiva version 9.0 software was used to acquire flow cytometry data and to perform cell sorting.

Data analysis

BD FlowJo version 10.8.1 software was used to gate and analyse flow cytometry data.

Microsoft Excel for Mac version 16.65 was used for data compilation and simple calculations (percentages, means, etc).

GraphPad Prism version 9.4.1 software was used to generate graphs and to perform statistical analysis, along with the online tool https://www.graphpad.com/quickcalcs/). Additional statistical analysis was also performed in R (version 4.1.1) using the following packages: lme4 version 1.1-30 and emmeans version 1.8.1-1.

Analysis of RNA sequencing data and integration with index-sort FACS data was performed in R (version 4.1.1) and GSEA software version 4.3.3. The code has been deposited to a public repository and is available on Zenondo under digital object identifier (DOI) 10.5281/zenodo.10925564 and include the following packages: abind (1.4-5), annotate (1.80.0), AnnotationDbi (1.64.1), askpass (1.2.0), assertthat (0.2.1), AUCell (1.24.0), backports (1.4.1), base64enc (0.1-3), batchelor (1.18.1), beachmat (2.18.0), beeswarm (0.4.0), BH (1.84.0-0), biglm (0.9-2.1), Biobase (2.62.0), BiocGenerics (0.48.1), BiocManager (1.30.22), BiocNeighbors (1.20.2), BiocParallel (1.36.0), BiocSingular (1.18.0), BiocVersion (3.18.1), Biostrings (2.70.1), bit (4.0.5), bit64 (4.0.5), bitops (1.0-7), blob (1.2.4), bluster (1.12.0), brio (1.1.4), broom (1.0.5), bslib (0.6.1), cachem (1.0.8), Cairo (1.6-2), callr (3.7.3), car (3.1-2), carData (3.0-5), caTools (1.18.2), cellranger (1.1.0), classInt (0.4-10), cli (3.6.2), clipr (0.8.0), colorspace (2.1-0), commonmark (1.9.0), conflicted (1.2.0), corrplot (0.92), cowplot (1.1.3), cpp11 (0.4.7), crayon (1.5.2), crosstalk (1.2.1), curl (5.2.0), cytolib (2.14.1), CytoML (2.14.0), data.table (1.14.10), DBI (1.1.3), dbplyr (2.4.0), DelayedArray (0.28.0), DelayedMatrixStats (1.24.0), deldir (2.0-2), desc (1.4.3), diffobj (0.3.5), digest (0.6.33), dotCall64 (1.1-1), dplyr (1.1.4), dqrng (0.3.2), dtplyr (1.3.1), e1071 (1.7-14), edgeR (4.0.6), ellipsis (0.3.2), evaluate (0.23), fansi (1.0.6), farver (2.1.1), fastDummies (1.7.3), fastmap (1.1.1),

fitdistrplus (1.1-11), flowClust (3.40.0), flowCore (2.14.1), flowViz (1.66.0), flowWorkspace (4.14.2), FNN (1.1.3.2), fontawesome (0.5.2), forcats (1.0.0), formatR (1.14), fs (1.6.3), furrr (0.3.1), futile.logger (1.4.3), futile.options (1.0.1), future (1.33.1), future.apply (1.11.1), gargle (1.5.2), generics (0.1.3), GENIE3 (1.24.0), GenomeInfoDb (1.38.5), GenomeInfoDbData (1.2.11), GenomicRanges (1.54.1), ggbeeswarm (0.7.2), ggcyto (1.30.0), ggplot2 (3.4.4), ggpointdensity (0.1.0), ggprism (1.0.4), ggrastr (1.0.2), ggrepel (0.9.5), ggridges (0.5.6), ggthemes (5.0.0), globals (0.16.2), glue (1.6.2), goftest (1.2-3), googledrive (2.1.1), googlesheets4 (1.1.1), gplots (3.1.3.1), graph (1.80.0), gridExtra (2.3), grr (0.9.5), GSEABase (1.64.0), gtable (0.3.4), gtools (3.9.5), haven (2.5.4), HDF5Array (1.30.0), here (1.0.1), hexbin (1.28.3), highr (0.10), hms (1.1.3), htmltools (0.5.7), htmlwidgets (1.6.4), httpuv (1.6.13), httr (1.4.7), ica (1.0-3), IDPmisc (1.1.21), ids (1.0.1), igraph (1.6.0), interp (1.1-6), IRanges (2.36.0), irlba (2.3.5.1), isoband (0.2.7), jpeg (0.1-10), jquerylib (0.1.4), jsonlite (1.8.8), KEGGREST (1.42.0), kernlab (0.9-32), knitr (1.45), labeling (0.4.3), lambda.r (1.2.4), later (1.3.2), latticeExtra (0.6-30), lazyeval (0.2.2), leiden (0.4.3.1), leidenbase (0.1.27), lifecycle (1.0.4), limma (3.58.1), listenv (0.9.0), lme4 (1.1-35.1), lmtest (0.9-40), locfit (1.5-9.8), lubridate (1.9.3), magrittr (2.0.3), Matrix (1.6-5), MatrixGenerics (1.14.0), MatrixModels (0.5-3), matrixStats (1.2.0), memoise (2.0.1), metapod (1.10.1), mime (0.12), miniUI (0.1.1.1), minqa (1.2.6), mixtools (2.0.0), modelr (0.1.11), munsell (0.5.0), ncdfFlow (2.48.0), nloptr (2.0.3), numDeriv (2016.8-1.1), openCyto (2.14.0), openssl (2.1.1), openxlsx (4.2.5.2), parallelly (1.36.0), patchwork (1.2.0), pbapply (1.7-2), pbkrtest (0.5.2), pbmcapply (1.5.1), pheatmap (1.0.12), pillar (1.9.0), pkgbuild (1.4.3), pkgconfig (2.0.3), pkgload (1.3.3), plogr (0.2.0), plotly (4.10.3), plyr (1.8.9), png (0.1-8), polyclip (1.10-6), praise (1.0.0), prettyunits (1.2.0), princurve (2.1.6), processx (3.8.3), progress (1.2.3), progressr (0.14.0), promises (1.2.1), proxy (0.4-27), ps (1.7.5), pscl (1.5.5.1), purrr (1.0.2), quantreg (5.97), R.methodsS3 (1.8.2), R.oo (1.25.0), R.utils (2.12.3), R6 (2.5.1), ragg (1.2.7), randomForest (4.7-1.1), RANN (2.6.1), rappdirs (0.3.3), RBGL (1.78.0), RColorBrewer (1.1-3), Rcpp (1.0.12), RcppAnnoy (0.0.21), RcppArmadillo (0.12.8.0.0), RcppEigen (0.3.3.9.4), RcppHNSW (0.5.0), RcppML (0.3.7), RcppProgress (0.4.2), RcppTOML (0.2.2), RCurl (1.98-1.14), readr (2.1.4), readxl (1.4.3), rematch (2.0.0), rematch2 (2.1.2), remotes (2.4.2.1), reprex (2.0.2), reshape2 (1.4.4), ResidualMatrix (1.12.0), reticulate (1.34.0), Rgraphviz (2.46.0), rhdf5 (2.46.1), rhdf5filters (1.14.1), Rhdf5lib (1.24.1), RhpcBLASctl (0.23-42), rlang (1.1.2), rmarkdown (2.25), ROCR (1.0-11), rprojroot (2.0.4), RProtoBufLib (2.14.0), rsample (1.2.0), RSpectra (0.16-1), RSQLite (2.3.4), rstatix (0.7.2), rstudioapi (0.15.0), rsvd (1.0.5), Rtsne (0.17), rvest (1.0.3), s2 (1.1.6), S4Arrays (1.2.0), S4Vectors (0.40.2), sass (0.4.8), ScaledMatrix (1.10.0), scales (1.3.0), scater (1.30.1), scattermore (1.2), scran (1.30.0), sctransform (0.4.1), scuttle (1.12.0), segmented (2.0-1), selectr (0.4-2), Seurat (5.0.1), SeuratObject (5.0.1), sf (1.0-15), shiny (1.8.0), SingleCellExperiment (1.24.0), sitmo (2.0.2), slam (0.1-50), slider (0.3.1), slingshot (2.10.0), snow (0.4-4), sourcetools (0.1.7-1), sp (2.1-2), spam (2.10-0), SparseArray (1.2.3), SparseM (1.81), sparseMatrixStats (1.14.0), spatstat.data (3.0-4), spatstat.explore (3.2-6), spatstat.geom (3.2-8), spatstat.random (3.2-2), spatstat.sparse (3.0-3), spatstat.utils (3.0-4), spData (2.3.0), spdep (1.3-1), speedglm (0.3-5), statmod (1.5.0), stringi (1.8.3), stringr (1.5.1), SummarizedExperiment (1.32.0), sys (3.4.2), systemfonts (1.0.5), tensor (1.5), terra (1.7-65), testthat (3.2.1), textshaping (0.3.7), tibble (3.2.1), tidyr (1.3.0), tidyselect (1.2.0), tidyverse (2.0.0), timechange (0.2.0), tinytex (0.49), tradeSeq (1.16.0), TrajectoryUtils (1.10.0), tzdb (0.4.0), units (0.8-5), utf8 (1.2.4), uuid (1.1-1), uwot (0.1.16), vctrs (0.6.5), vipor (0.4.7), viridis (0.6.4), viridisLite (0.4.2), vroom (1.6.5), waldo (0.5.2), warp (0.2.1), withr (2.5.2), wk (0.9.1), xfun (0.41), XML (3.99-0.16), xml2 (1.3.6), xtable (1.8-4), XVector (0.42.0), yaml (2.3.8), zip (2.3.0), zlibbioc (1.48.0), zoo (1.8-12), base (4.3.2), boot (1.3-28.1), class (7.3-22), cluster (2.1.4), codetools (0.2-19), compiler (4.3.2), datasets (4.3.2), foreign (0.8-85), graphics (4.3.2), grDevices (4.3.2), grid (4.3.2), KernSmooth (2.23-22), lattice (0.21-9), MASS (7.3-60), Matrix (1.6-1.1), methods (4.3.2), mgcv (1.9-0), nlme (3.1-163), nnet (7.3-19), parallel (4.3.2), rpart (4.1.21), spatial (7.3-17), splines (4.3.2), stats (4.3.2), stats4 (4.3.2), survival (3.5-7), tcltk (4.3.2), tools (4.3.2), utils (4.3.2).

For manuscripts utilizing custom algorithms or software that are central to the research but not yet described in published literature, software must be made available to editors and reviewers. We strongly encourage code deposition in a community repository (e.g. GitHub). See the Nature Portfolio guidelines for submitting code & software for further information.

Data

Policy information about availability of data

All manuscripts must include a data availability statement. This statement should provide the following information, where applicable:

- Accession codes, unique identifiers, or web links for publicly available datasets
- A description of any restrictions on data availability
- For clinical datasets or third party data, please ensure that the statement adheres to our policy

Data availability
Source data for all figures related to FACS analysis and in vitro lineage potentials is available in the online version of the paper. Additional relevant information and material will be available from the corresponding authors upon request (j.carrelha@imperial.ac.uk / sten.eirik.jacobsen@ki.se). RNA sequencing data have been deposited to the public repository ArrayExpress under accession number E-MTAB-13935.

Code availability
Code for RNA sequencing analysis and statistical analysis of fate mapping data has been deposited to the public repository Zenodo with digital object identifier (DOI): 10.5281/zenodo.10925564.

Research involving human participants, their data, or biological material

Policy information about studies with human participants or human data. See also policy information about sex, gender (identity/presentation), and sexual orientation and race, ethnicity and racism.

Reporting on sex and gender	N/A
Reporting on race, ethnicity, or other socially relevant groupings	N/A
Population characteristics	N/A
Recruitment	N/A
Ethics oversight	N/A

Note that full information on the approval of the study protocol must also be provided in the manuscript.

Field-specific reporting

Please select the one below that is the best fit for your research. If you are not sure, read the appropriate sections before making your selection.

☒ Life sciences ☐ Behavioural & social sciences ☐ Ecological, evolutionary & environmental sciences

For a reference copy of the document with all sections, see nature.com/documents/nr-reporting-summary-flat.pdf

Life sciences study design

All studies must disclose on these points even when the disclosure is negative.

Sample size	Based on our previous knowledge with the experimental setup, the required number of single HSC transplanted mice was estimated with the goal of generating enough reconstituted mice with each of the reconstitution categories of interest to be used in multiple downstream experiments. Being P-HSC ~5-10% of Vwf-tdTomato+ (Carrelha et al. 2018) and Multi-HSC ~ 70% Vwf-tdTomato- (see ED Fig.1), we single cell transplanted equal numbers of Vwf-dTom+ and Vwf-dTom- HSCs in each cohort of 40-60 mice to obtain beetween 2-10 mice per experimental cohort. RNA sequencing: sample sizes were not predetermined using statistical analysis, but cell type comparisons or methods comparisons typically contained hundreds of cells per group. One 384 well plate was collected per mouse. At least 7 mice were included per reconstitution category/group for experiments, and at least 3 mice when validating results from RNA sequencing. Considering the preserved patterns of gene expression for cells within individual biological replicates and between biological replicates with the same reconstitution pattern, this sample size was considered sufficient for the study. As sample size was not predetermined for other experiments, a similar strategy as above was applied when considering a sufficient sample size.
Data exclusions	Blood lineage reconstitution patterns upon single HSC transplantation were defined as described in detail in Results and Methods. We included for further analysis all mice reconstituted with single cells fulfilling the described definition of being Vwf+ P-restricted, Vwf+ P-HSCs, or Vwf- Multi-HSCs without P, PE, or PEM bias.<br>In secondary transplantations, primary donors that failed to reconstitute secondary recipients with a positive control population were excluded from further analysis (i.e., lack of output from a population of interest was not considered robust unless accompanied by positive output from the positive control population).<br>In order not to include mice with incomplete recombination in fate mapping experiments, Flt3Cre and VavCre mice with <98% of reporter labelling in erythroid cells, myeloid cells, B cells and T cells and/or for Flt3Cre mice if <98% of reporter labelling inFLT3 positive bone marrow progenitor cells were excluded from analysis (even if possessing the genotype of interest).<br>Single-cell RNA-seq data were filtered according to established quality control criteria for removing technically failed cells. Cutoffs are listed where appropriate.<br>For anti-CD42b experiments mice, Sysmex analysis at 3 days post injection were used to validate the platelet depletion. When Sysmex analysis could not validate the platelet depletion due to, for example, clot formation during blood collection or suboptimal intravenous injections, mice were excluded from downstream analysis.
Replication	Each type of experiment was repeated at least twice (platelet depletion, CP treatment in VavCre mice) or more (34 replicates for single cell transplantations), by multiple investigators, across multiple dates, and using mice from multiple litters. All experimental replicates showed very little inter-experiment variability. All RNA sequencing experiments were performed across hundreds of individual cells and using several mice transplanted both at Karolinska Institute and at University of Oxford.
Randomization	For in vivo single cell transplantation experiment randomization was not required as we are unable to predict the reconstitution pattern from a single HSC prior to transplantation. For single cell sorting for transplantation and for RNA sequencing, random individual cells of each population were FACS sorted into wells of microplates according to a predefined sort layout. For treatment with CP, 5FU and CD42b antibody the mice were randomly allocated to PBS and treatment with agents.
Blinding	For in vivo single cell transplantation experiment blinding was not required as we are unable to predict the reconstitution pattern from a single HSC prior to transplantation. With the exception chemotherapy treatment (CP and 5FU) where the mice had to be allocated to different cages during treatment in order to avoid unspecific effects in untreated cage-mates, the investigators were blinded to condition or treatment by the use of generic mouse ID numbers which did not reveal the experimental group.

Reporting for specific materials, systems and methods

We require information from authors about some types of materials, experimental systems and methods used in many studies. Here, indicate whether each material, system or method listed is relevant to your study. If you are not sure if a list item applies to your research, read the appropriate section before selecting a response.

Materials & experimental systems

n/a	Involved in the study
☐	☒ Antibodies
☒	☐ Eukaryotic cell lines
☒	☐ Palaeontology and archaeology
☐	☒ Animals and other organisms
☒	☐ Clinical data
☒	☐ Dual use research of concern
☒	☐ Plants

Methods

n/a	Involved in the study
☒	☐ ChIP-seq
☐	☒ Flow cytometry
☒	☐ MRI-based neuroimaging

Antibodies

Antibodies used	Antibody details in Supplementary Table 6 where dilutions, catalog number and lot number are listed for each antibody used in the study.
Validation	The antibodies used have been previously validated in the mouse haematopoietic system for the same applications as in this study. In addition, we validated all antibodies (and each batch of each antibody) by titration with relevant cells, using staining panels that included antibodies for negative and positive control antigens.

Animals and other research organisms

Policy information about studies involving animals; ARRIVE guidelines recommended for reporting animal research, and Sex and Gender in Research

Laboratory animals	Vwf-tdTomatotg/+ Gata1-eGFPtg/+ mice, C57BL/6OlaHsd and C57BL/6JrJ background, 7-14 weeks old.<br>Flt3Cretg/+ R26Tom/+(Ai9) Vwf-eGFPtg/+ Gata1-eGFPtg/+ mice, C57BL/6OlaHsd and C57BL/6JrJ background, 7-14 weeks old.<br>Flt3Cretg/+ R26Tom/+(Ai14) mice, C57BL/6JrJ background, 7-11 weeks old.<br>VavCretg/+ R26Tom/+(Ai14) mice, C57BL/6JrJ background, 8-23 weeks.<br>Wildtype CD45.1 B6.SJL-Ptprca Pepcb/BoyJ and B6.SJL-Ptprca Pepcb/BoyCrl, 7-16 weeks old.<br>Mice were housed in individually ventilated cages at the Oxford JR facility, with 12/12h light/dark cycle, at 19-24 °C, and humidity 45-65% and at the Karolinska Institute KM facilities, with 12/12h light/dark cycle, 22 ± 1°C and 50% humidity.
Wild animals	No wild animal were used in the study
Reporting on sex	In the Flt3Cre mouse model, the Cre+ genotype is restricted to males and, therefore, only males were used. Donor mice, recipient mice and competitor cells were sex-matched in transplantation experiments (i.e. all females or all males).
Field-collected samples	No field-collection samples were used in the study.
Ethics oversight	Animal experiments performed at University of Oxford were approved by the Oxford Clinical Medicine Ethical Review Committee, and at the Karolinska Institutet by the regional review committee for animal ethics; Stockholms djurförsöksetiska nämnd. All experimental procedures and mouse breeding were performed in accordance with UK Home Office regulations and Swedish Jordbruksverket regulations.

Note that full information on the approval of the study protocol must also be provided in the manuscript.

Plants

Seed stocks	N/A
Novel plant genotypes	N/A
Authentication	N/A

Flow Cytometry

Plots

Confirm that:

☒ The axis labels state the marker and fluorochrome used (e.g. CD4-FITC).

☒ The axis scales are clearly visible. Include numbers along axes only for bottom left plot of group (a 'group' is an analysis of identical markers).

☒ All plots are contour plots with outliers or pseudocolor plots.

☒ A numerical value for number of cells or percentage (with statistics) is provided.

Methodology

Sample preparation	Leg, pelvis, sternum, and spine bones were collected immediately after culling mice and crushed with pestle and mortar. Blood samples were collected from live mice by tail vein bleeding into Lithium-Heparin tubes, or by cardiac puncture immediately after culling mice. Bone marrow and peripheral blood were prepared into single cell suspension in PBS supplemented with 1-5% fetal calf serum and 2 mM EDTA. All samples were incubated with purified CD16/32 (Fc-Block) prior to staining with monoclonal antibodies, unless the analysis used conjugated CD16/32 for analysis of myeloid progenitor populations. Details in Methods.
Instrument	BD FACSAriaII, BD FACSAriaIII, BD FACSAria Fusion, BD LSRII, BD LSR Fortessa, BD LSR Fortessa X-20.
Software	BD FACSDiva version 9.0 software was used to acquire flow cytometry data and to perform cell sorting. BD FlowJo version 10.8.1 software was used to gate and analyse flow cytometry data.
Cell population abundance	At the beginning and end of sorting sessions, a test sort and immediate purity analysis in the same sorter was performed for the population of interest (or from a parent gate for rare populations). Typically 100-300 cells were sorted and purity upon reanalysis was ≥95% percent when factoring in all impurities in all hierarchical gates.<br>Additional considerations in single cell sort experiments: index sorting recorded the cell surface expression of markers in each single cell sorted; accurate single cell deposition into plates was validated using 488 nm fluorescent beads before and after sorting sessions.
Gating strategy	FSC-A/SSC-A was used for gating mononuclear cells. SSC-H/SSC-W and/or FSC-A/FSC-H were used to exclude doublets and select singlets. DAPI-positive or 7AAD-positive cells were gated out to exclude non-viable cells. cKIT/LIN was used for gating out Lineage-positive cells in bone marrow, in order to focus analysis on early progenitors. Whenever possible, the phenotypic definition of each population included both positive and negative markers. Fluorescence-minus-one controls (FMOs) were recorded but, as much as possible, the boundaries of negative and positive populations were set based on internal negative control populations within each sample. Specific gating strategies are outlined in Methods and exemplified in Figures and Extended Data Figures.

☒ Tick this box to confirm that a figure exemplifying the gating strategy is provided in the Supplementary Information.
